# Measuring Coverage in MNCH: Validating Women’s Self-Report of Emergency Cesarean Sections in Ghana and the Dominican Republic

**DOI:** 10.1371/journal.pone.0060761

**Published:** 2013-05-07

**Authors:** Özge Tunçalp, Cynthia Stanton, Arachu Castro, Richard Adanu, Marilyn Heymann, Kwame Adu-Bonsaffoh, Samantha R. Lattof, Ann Blanc, Ana Langer

**Affiliations:** 1 Johns Hopkins Bloomberg School of Public Health Baltimore, Maryland, United States of America; 2 Harvard Medical School, Harvard University, Boston, Massachusetts, United States of America; 3 School of Public Health, University of Ghana, Accra, Ghana; 4 Korle Bu Teaching Hospital, University of Ghana, Accra, Ghana; 5 Harvard School of Public Health, Harvard University, Boston, Massachusetts, United States of America; 6 Population Council, New York, New York, United States of America; Kings College London, United Kingdom, in consultation with Carla AbouZahr, independent consultant, health statistics and policy

## Abstract

**Background:**

Cesarean section is the only surgery for which we have nearly global population-based data. However, few surveys provide additional data related to cesarean sections. Given weaknesses in many health information systems, health planners in developing countries will likely rely on nationally representative surveys for the foreseeable future. The objective is to validate self-reported data on the emergency status of cesarean sections among women delivering in teaching hospitals in the capitals of two contrasting countries: Accra, Ghana and Santo Domingo, Dominican Republic (DR).

**Methods and Findings:**

This study compares hospital-based data, considered the reference standard, against women’s self-report for two definitions of emergency cesarean section based on the timing of the decision to operate and the timing of the cesarean section relative to onset of labor. Hospital data were abstracted from individual medical records, and hospital discharge interviews were conducted with women who had undergone cesarean section in two hospitals. The study assessed sensitivity, specificity, and positive predictive value of responses to questions regarding emergency versus non-emergency cesarean section and estimated the percent of emergency cesarean sections that would be obtained from a survey, given the observed prevalence, sensitivity, and specificity from this study. Hospital data were matched with exit interviews for 659 women delivered via cesarean section for Ghana and 1,531 for the Dominican Republic. In Ghana and the Dominican Republic, sensitivity and specificity for emergency cesarean section defined by decision time were 79% and 82%, and 50% and 80%, respectively. The validity of emergency cesarean defined by operation time showed less favorable results than decision time in Ghana and slightly more favorable results in the Dominican Republic.

**Conclusions:**

Questions used in this study to identify emergency cesarean section are promising but insufficient to promote for inclusion in international survey questionnaires. Additional studies which confirm the accuracy of key facility-based indicators in advance of data collection and which use a longer recall period are warranted.

*This paper is part of the* PLOS Medicine *“Measuring Coverage in MNCH” Collection.*

## Introduction

Cesarean section rates are rising in many low- and middle-income countries. For the first time, the World Health Organization’s (WHO) *World Health Statistics 2012* reports a global cesarean section rate (16%) that exceeds the frequently used upper recommended limit of 15% [1], [2]. Even in a low-income country like Bangladesh, recent data show the cesarean section rate increased from 3% to 12% between 2001 and 2010 [3]. Some middle-income Latin American and Asian countries report rates between 30% and 46%, and the cesarean section rate for upper-middle-income countries has surpassed that of high-income countries (31% and 28% respectively) [2]. Extreme socio-economic disparities in access to cesarean section exist within low-income countries as well. Women in the wealthiest households often have rates above 20%, whereas among the poorest households in many countries, cesarean section rates are less than one percent [4].

High and rising national rates indicate cause for concern, but provide no information on why or how these rates are changing or whether the increase is associated with any health gains. Likewise, very low rates, as seen in much of sub-Saharan Africa, provide no assurance that cesarean sections are serving women in greatest need.

Currently, cesarean section is the only surgery for which we have nearly global population-based data [2], [5], as a result of the Demographic and Health Surveys (DHS) and UNICEF’s Multiple Indicator Cluster Surveys (MICS). However, few surveys in low-income countries have incorporated questions that go beyond mode of delivery [6].

Although large-scale surveys provide the majority of global data on cesarean section, the question on cesarean section has not been validated. One study assessing the reliability of self-reported cesarean section rates in the DHS in six low-income countries showed that self-reported cesarean section rates were consistently higher than hospital-based cesarean section data applied to population-based births. However, in three quarters of the 31 sub-national observations assessed, hospital-based rates fell within 95% confidence intervals of the survey-based estimates. The differences between the two were often less than one percentage point [7]. It is not surprising that reliability of self-reported cesarean section is high since women are unlikely to forget or fabricate having undergone cesarean section.

In response to the need for more in-depth information related to cesarean section, the Maternal Health Task Force and the Child Health Epidemiology Reference Group at Johns Hopkins University sponsored a meeting in February 2010 in Baltimore, Maryland for maternal health researchers and program managers to propose an expanded list of indicators related to cesarean section [8]. Their top recommendation and the impetus for this study was the need to validate an indicator of emergency cesarean section which could be obtained from surveys of women of reproductive age.

Numerous definitions of emergency cesarean section exist, each of which identify a somewhat different group of women. A Medline search on emergency cesarean section from 1982 through 2007 by Schauberger and Chauhan [9] reported 28 studies which used at least 12 definitions based on varying criteria including: decision for cesarean section was made in labor, not scheduled, severe maternal/fetal complications (complications were specified in some but not all studies), immediate threat to mother/fetal life, timeliness from decision to incision or delivery, and various combinations of the above-mentioned criteria. In eight studies, no definition was provided. In a recent systematic review of cesarean section classification systems, Torloni and colleagues [10] identified nine classification systems (four based on indications and five based on various definitions of “urgency”) that do not always use the term “emergency” but are similar in concept; for example: absolute maternal indication, obligatory, extreme emergency, and crash. In almost half of these studies, the classification system was designed for use in high-income countries with sophisticated record keeping.

This study, part of the *PLOS Medicine* “Measuring Coverage in MNCH” Collection, has three objectives. The first is to validate self-reported data on emergency cesarean section among a sample of women who delivered by cesarean section. Two definitions of emergency cesarean section are tested. Cesarean section *by decision time* refers to a cesarean section for which the decision to perform the operation is made after the onset of labor. Cesarean section *by operation time* refers to a cesarean section performed after the onset of labor. Both indicators are dichotomous. We test two definitions because (1) in low-income settings, emergency cesarean section based on decision time may more accurately reflect the chronology of events than operation time given inadequate staffing and resources which often lead to delayed care; and (2) the timing of the operation relative to labor may be easier for women to report. To increase generalizability, large hospitals in two contrasting countries were selected for this study: Ghana and the Dominican Republic.

The second objective of the study is to estimate the percentage of emergency cesarean sections that would be obtained from a population-based survey, given the assessment of sensitivity and specificity from this study. The third objective is to identify characteristics of women who accurately report the status of their delivery by cesarean section.

### Contrasting Countries: Ghana Versus the Dominican Republic

In Ghana, maternal mortality is high at 378 per 100,000 births in 2007 [11]. Skilled attendance at birth in Ghana has increased over the past 20 years from 41% to 60% [12], most of which has occurred since 2003 when the Ghana Health Service began fee exemption for delivery services [13]. According to the Ghana DHS survey, the cesarean section rate increased from 4.5% to 6.4% between 1990 and 2005, with greater than 10-fold differentials in the rate by wealth quintile. As of 2005, the cesarean section rate for 40% of the population was under the WHO recommended minimum of 5%, and under 1% for the poorest quintile [12]. In contrast, the Dominican Republic is a country with nearly universal coverage of antenatal care and institutional delivery (>95%) [14], high maternal mortality compared to countries of similar income (179 per 100,000 live births between 2004 and 2008) [8], and rapidly increasing cesarean section rates. Between 1990 and 2006, the cesarean section rate in the Dominican Republic doubled from 22% to 44% [12].

## Methods

The study was conducted in two hospitals. Korle-Bu Hospital is one of the largest teaching hospitals in Ghana, situated in the capital, Accra. It is a tertiary referral center with 10,000 annual deliveries and a cesarean section rate of 30%. In the Dominican Republic, the study was conducted at the Maternity Hospital Nuestra Señora de la Altagracia, the national referral maternity hospital and a teaching hospital, in the capital, Santo Domingo. It is a tertiary level hospital with approximately 18,000 deliveries annually and a cesarean section rate of 33% [15]. Both of the facilities used partographs as routine practice during labor and delivery, although their use might not be consistent at times.

For the first objective, sensitivity, specificity, and positive predictive value of indicators related to caesarean section were calculated from women’s responses to questions in the exit interview compared against hospital-based data (considered the reference standard). Area under the receiver operating characteristic curve (AUC) was estimated for each variable to compare overall validity for each indicator. Thus, research assistants undertook two data collection activities: (1) they abstracted data from the surgical and delivery room registers, individual case notes, and, occasionally, inquiries to the physician; and (2) they conducted face-to-face interviews just prior to hospital discharge of all women who had undergone cesarean section in each hospital. In Ghana, interviews were conducted in Twi and English. In the Dominican Republic, interviews were conducted in Spanish and Haitian Creole.

All women undergoing cesarean section were eligible for the study. Written informed consent was obtained upon admission to the hospital. Data were collected in Accra from June to August 2011, and from August to November 2011 in Santo Domingo. The following descriptive information was also collected: characteristics of the woman and the provider/patient communication she experienced during her hospital stay (from the exit interview), and hospital characteristics such as the patient/provider ratio, volume of births, and deliveries by cesarean section (from hospital administrative data). The formulation of the questions assessed in this study is summarized in Box 1 (with Spanish version in Text S1), along with the two definitions of emergency cesarean section.

**Box 1. Questions Used in the Exit Interview**

**GENERAL BACKGROUND QUESTIONS:**

- Previous to this pregnancy, have you ever had a cesarean section?
  1. If YES, previous to this pregnancy, how many cesarean deliveries have you had?
- Other than that, have you ever had any surgery/operation in your pelvic area?
  1. If YES, what was the surgery/operation?

**CURRENT DELIVERY:**

- Were you planning to deliver at Korle-Bu Teaching Hospital/Maternidad Altagracia?
  1. If NO, where were you planning on delivering?
- Were you transferred from another facility?
  1. IF YES, from where?
  2. What was the reason for your transfer?
- What kind of delivery have you had here at Korle-Bu/Maternidad Altagracia?
- What was the reason for your operation during your delivery? Choose the reason that best applies to your situation (includes a write-in option for other reasons)
- When was the decision made for you to have a cesarean/operation?
- Whose idea was it for you to have a cesarean/operation? Please select the choice that best describes whose idea it was (includes a write-in option for other).
- Why did you request the cesarean?
- Who told you that you were having an operation/−cesarean section?
- Did you go into labor by yourself/spontaneously?
- Did a health care provider give you a medication or drip to START your labor?
- Did you get a cesarean section BEFORE your labor pains began?
- How many weeks were you when you delivered?
- Was the baby born early? Was the baby born on time (at term)?

**EMERGENCY CESAREAN SECTION QUESTIONS**

**Decision Time:**

- When was the decision made for you to have a cesarean/operation?
  1. During antenatal clinic visits
  2. Before the labor pains began
  3. After labor pains began
  4. Don’t know

**Operation Time:**

- Did you go into labor by yourself/spontaneously?
  1. Yes
  2. No
  3. Don’t Know
- Did a health care provider give you a medication or drip to START your labor?
  1. Yes
  2. No
  3. Don’t Know
- Did you get a cesarean section BEFORE your labor pains began?
  1. Yes
  2. No
  3. Don’t Know

**EMERGENCY CESAREAN SECTION DEFINITIONS**

- Emergency Cesarean Section defined by Decision Time:
  1. When was the decision made for you to have a cesarean?

Answer: After labor pains began

- Emergency Cesarean Section defined by Operation Time:
  1. Did you go into labor by yourself/spontaneously?

Answer: Yes

  1. Did a health care provider give you a medication or drip to START your labor?

Answer: Yes/No (depending on the answer to the first question)

  1. Did you get a cesarean section BEFORE your labor pains began?

Answer: No

The method used for the second objective replicates methods used by Ronsmans and colleagues when assessing obstetric complications in Indonesia [16]. Using the equation below from Vecchio [17], sensitivity and specificity estimates from the validation study were used to calculate the prevalence of emergency cesarean section and other indicators of interest that would be obtained from a population-based survey, using the following equation:
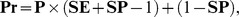
where Pr is the estimate of survey-based prevalence, P is the hypothetical “true” prevalence in the population, SE is sensitivity, and SP is specificity. Results regarding the estimated population-based emergency cesarean section rate were expressed as an inflation factor (IF), that is, as an over- or under-estimation factor relative to the “true” rate. This equation is the mathematical equivalent of the ratio of Test to Actual Positives (TAP ratio) [18], which has been utilized in a number of papers in this Collection. Of note, two assumptions underlie this calculation:

1. Self-report of cesarean section is valid. Thus, the sample is restricted to women who had undergone cesarean. This sample is appropriate for a validation study of emergency cesarean section because in a survey questionnaire, only women who had delivered by cesarean would be asked questions regarding the characteristics of the procedure.
2. Results from interviews at hospital discharge are generalizable to survey-based responses about events up to three years prior to the survey; that is, we assume that poor recall of an event as major as pelvic surgery is low.

Unadjusted logistic regression was used to assess the third objective, with accurate self-report of emergency cesarean section as the dependent variable and women’s characteristics as the independent variables.

Sample size for the study was calculated before the data collection and was based on an assumption of 80% sensitivity, a Type 1 error at 5% for a two-tailed test, ±5% precision and the true proportion of cesarean sections that are emergency cesarean sections at 30% in Ghana and 5% in Dominican Republic. Based on these assumptions, the target sample size was 450 women who had been delivered by cesarean section in the Ghanaian site and 1,460 in the Dominican Republic.

Ethical approval for the study in Ghana was provided by the Institutional Review Board of Korle-Bu Teaching Hospital, University of Ghana Medical School, College of Health Sciences, Accra, Ghana. The Harvard School of Public Health and the National Council of Bioethics of the Dominican Republic approved the study in the Dominican Republic.

## Results

### Study Population

**Figure 1:**
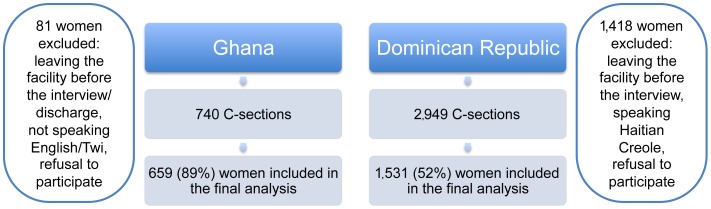
Flowchart of participation in Ghana and the Dominican Republic.

In Ghana, 740 women were delivered by cesarean section during the study period, of which 89% (659 women) were interviewed prior to hospital discharge (Figure 1). Of 81 exit interviews that were missed, 64 were women who left the hospital before the interview, 15 did not speak English or Twi, one left the facility before her discharge, and one refused participation. The median number of days between the operation and the interview was 3 days (interquartile range [IQR] 2–3). In the Dominican Republic, 2,949 women were delivered by cesarean section during the study, of which 52% (1531 women) were interviewed before hospital discharge and included in the analysis. Twelve women refused participation, 92 women interviewed in Haitian Creole were excluded, and the rest (1,314 women) left the hospital before they could be interviewed. Factors that limited the Dominican Republic team’s ability to invite the women to participate in the study included the lack of availability of medical files for review, the movement of patients within the hospital, and the early discharge practices of the hospital. The median number of days between the operation and the interview was one day (IQR 1–2).

### Characteristics of the Women in Ghana and the Dominican Republic

Table 1 presents the characteristics of the two study populations (as reported in exit interviews), which differ substantially. In Ghana as compared to the Dominican Republic, mean age and parity were higher and education was lower. The population in Ghana was more rural than in the Dominican Republic, as expected, and the distribution by religion varied. Proportions of women with previous cesarean sections were similar across the two populations (35% in Ghana and 38% in the Dominican Republic). Given the large difference in cesarean section rates in the two countries, a lower previous cesarean section rate in Ghana might have been expected. The rate of other pelvic surgery was low in both samples (4.7% in Ghana and 2.7% in the Dominican Republic).

**Table 1:** Background characteristics of the study population based on women’s exit interviews.

Sociodemographic Characteristics	Ghana (N = 659)	Dominican Republic (N = 1,531)	*p*-Value^a^
**Age, years**			0.0001
15–19	16 (2.4)	416 (27.2)	
20–24	89 (13.5)	501 (32.7)	
25–29	176 (26.7)	332 (21.7)	
30–34	219 (33.2)	188 (12.3)	
35–39	127 (19.3)	76 (4.9)	
40–49	32 (4.9)	18 (1.2)	
**Education**			0.0001
None	48 (7.3)	26 (1.7)	
Primary	87 (13.2)	402 (26.3)	
Secondary	433 (65.7)	861 (56.2)	
Tertiary	91 (13.8)	242 (15.8)	
**Religion**			0.0001
Christian	566 (85.9)	1,065 (69.6)	
Muslim	92 (13.9)	0 (0.0)	
Other	1 (0.2)	22 (1.4)	
No religion	0 (0.0)	444 (29.0)	
**Marital Status**			0.692
Married/cohabitation	556 (84.4)	1,290 (84.3)	
Single	100 (15.1)	229 (15.0)	
Divorced/separated	3 (0.5)	12 (0.7)	
**Residence**			0.0001
Urban	520 (78.9)	1,396 (91.2)	
Rural	136 (20.6)	131 (8.6)	
Don’t know	3 (0.5)	4 (0.2)	
**Obstetric history**			
* Number of pregnancies, mean (SD)*	2.81 (1.59)	3.01 (1.83)	0.0158
* Number of previous deliveries, mean (SD)*	2.34 (1.39)	2.12 (1.32)	0.0003
* Previous cesarean section*			0.167
No	426 (64.6)	942 (61.5)	
Yes	233 (35.4)	589 (38.5)	
* Previous pelvic surgery (other than cesarean section)*			0.021
No	628 (95.3)	1,490 (97.3)	
Yes	31 (4.7)	41 (2.7)	
**Current pregnancy**			
* Gestational age at delivery (weeks)*			0.001
<35	21 (3.2)	141 (9.2)	
35–37	52 (7.9)	262 (17.1)	
38–40	130 (19.7)	797 (52.1)	
41–43	38 (5.8)	229 (14.9)	
Don’t know	418 (63.4)	102 (6.7)	
* Gestational age in terms*			0.001
Preterm	180 (27.3)	263 (17.2)	
Term	318 (48.2)	1,230 (80.3)	
Post-term	125 (19.1)	13 (0.9)	
Don’t know	36 (5.4)	25 (1.6)	
* Multiple pregnancy*			0.24
Single	623 (94.5)	1,465 (95.7)	
Multiple	36 (5.5)	66 (4.3)	
* Delivery plan*			0.001
Home	14 (2.1)	0 (0.0)	
Study hospital	280 (42.5)	1,201 (78.5)	
Other facility	365 (55.4)	330 (21.5)	
* Referral status*			0.001
No	151 (22.9)	1,139 (74.4)	
Yes	508 (77.1)	392 (25.6)	
**Cesarean-section indicators**			
* Reporting of cesarean section*			n/a
No	0 (0)	0 (0)	
Yes	659 (100)	1,531 (100)	
* Reporting of time of cesarean section decision*			0.001
During antenatal visits	208 (31.6)	751 (49.1)	
Before labor	169 (25.6)	165 (10.8)	
After onset of labor	276 (41.9)	597 (38.9)	
Don’t know	6 (0.9)	18 (1.2)	
* Reporting of time of cesarean section*			0.001
Spontaneous labor	328 (49.8)	1,047 (68.4)	
Induced labor	35 (5.3)	5 (0.33)	
Cesarean section before labor	278 (42.2)	359 (23.4)	
Don’t know	18 (2.7)	120 (7.8)	
**Communication**			
* Cesarean section decision maker*			0.001
The doctor	591 (89.7)	1,510 (98.6)	
The woman	35 (5.3)	10 (0.6)	
Other	6 (0.9)	0 (0)	
Don’t know	27 (4.1)	11 (0.7)	
* Cesarean section information*			0.001
Doctor	571 (86.6)	1,393 (96.8)	
Nurse/midwife	46 (7.0)	10 (0.7)	
No one	38 (5.8)	33 (2.3)	
Other	4 (0.6)	3 (0.2)	

a Pearson’s Chi-square tests and/or Yates correction for continuity (when necessary) are used for bivariate and categorical variables. T-tests are used for continuous variables.

The data on delivery plan and referral status best illustrate the difference in case mix between the two hospitals. In the Dominican Republic, nearly four fifths of women planned on delivering at Altagracia Hospital and one quarter of women report being referred to this hospital. In contrast, in Ghana 42% of women planned on delivering at Korle Bu Hospital and over three quarters of women were referred and transferred. Although both hospitals are large urban teaching hospitals, Korle Bu appears to be used more frequently as a referral hospital than Altagracia, suggesting that complicated deliveries likely represent a higher percentage of deliveries in Korle Bu than in Altagracia. This may partially explain the similar rates of previous cesarean sections in the two hospitals.

In both populations, 100% of women reported having undergone a cesarean section. In Ghana, 57% of women reported that the decision for delivery via cesarean was made before the onset of labor (nearly half of which during antenatal care visits); 42% reported that the decision was made after the onset of labor. In the Dominican Republic, women reported that the decision for a cesarean section was made before the onset of labor in 60% of cases, of which more than four fifths were made during antenatal care visits; in 39% of the cases, the decision was made after the onset of labor.

According to women’s report, the onset of labor also varied across the two populations. Among women in Ghana, half of women had a spontaneous onset of labor, 5.3% of women had their labor induced, and 42% of women underwent cesarean section before the onset of labor. In the Dominican Republic, two-thirds of women had a spontaneous onset of labor, there were almost no inductions (0.3%), and 23% underwent cesarean section prior to the onset of labor.

The majority of the women in both of the study populations reported that the decision to perform a cesarean section was made by a doctor and that the doctor informed them about this decision.

### Validation of Cesarean Section Indicators

Table 2 presents the prevalence, sensitivity, specificity, positive predictive value, and AUC and IF for emergency cesarean defined by decision time and by operation time relative to the onset of labor. It should be noted that information on these indicators was mainly collected from the patient files in both of our study settings, represented as a percentage within the study population. For ten cases in Ghana (1.5%) and 36 cases in the Dominican Republic (2.3%), this was supplemented by information requested from the medical staff.

**Table 2:** Validation assessment of cesarean section indicators.

Ghana (N = 659)	% (Within the Study Population)*	Sensitivity (95% CI)	Specificity (95% CI)	AUC Point Estimate (95% CI)	Positive Predictive Value (%)	Population-Based Survey Estimate (%)	IF
Previous cesarean section	36.9	95 (91–97)	98 (97–99)	0.96 (0.95–0.98)	97	37	0.98
Emergency cesarean section by decision time	39.8	79 (73–83)	82 (78–85)	0.80 (0.77–0.83)	74	42	1.06
Emergency cesarean section by the operation time (single question)	48.8	84 (80–88)	68 (63–73)	0.79 (0.72–0.79)	74	57	1.18
Emergency cesarean section by the operation time (three–question algorithm)	49.4	84 (80–88)	70 (65–75)	0.77 (0.74–0.80)	72	57	1.15
Spontaneous labor	42.2	85 (80–89)	73 (69–78)	0.79 (0.76–0.82)	70	51	1.21
Induced labor	7.2	37 (23–51)	97 (96–98)	0.67 (0.59–0.74)	49	5	0.76
Cesarean section before labor	50.5	70 (65–75)	84 (80–88)	0.77 (0.74–0.80)	82	43	0.86
**Dominican Republic (N = 1,531)**							
Previous cesarean section	38.2	96 (94–98)	97 (96–98)	0.96 (0.96–0.98)	95	38.5	1.01
Emergency cesarean Section by decision time	64.7	50 (47–53)	80 (77–83)	0.65 (0.62–0.67)	82	39	0.61
Emergency cesarean section by the operation time (single question)	66.0	83 (80–85)	53 (48–57)	0.67 (0.65–0.70)	79	71	1.07
Emergency cesarean section by the operation time (three question algorithm)	67.0	88 (86–90)	53 (48–57)	0.70 (0.68–0.73)	77	74	1.11
Spontaneous labor	62.0	89 (87–91)	51 (46–55)	0.70 (0.68–0.72)	75	74	1.19
Induced labor	5.0	1.4 (−24–27)	99.7 (0.99–1)	0.50 (0.49–0.52)	20	0.3	0.07
Caesarean section before labor	33.0	53 (48–57)	88 (86–90)	0.70 (0.68–0.73)	68	26	0.77

* The percentages used in this column are based on the data collected from the reference standard (patient records).

In Ghana, emergency cesarean section defined by decision time shows sensitivity and specificity of approximately 80% (79% and 82%, respectively) and an IF of 1.06. Emergency cesarean section defined by decision time in the Dominican Republic had similar specificity (80%), yet lower sensitivity (50%), leading to an IF suggesting almost 40% underestimation in a population-based survey (0.61). Given the higher prevalence of this indicator in Dominican Republic, positive predictive value was higher in Dominican Republic than in Ghana (82% versus 74%).

Emergency cesarean section by operation time had sensitivity of 84%, specificity of 68%, and IF of 1.18 in Ghana; in the DR, sensitivity was 83%, specificity 53% and the IF was 1.07. Positive predictive value varied between 72% and 79% for both of the settings, slightly higher in the Dominican Republic. For exploratory purposes, the definition of operation time was refined by using the responses to two additional survey questions, which first specified that the woman did experience labor. Thus, women with emergency cesarean section were defined as: (1) those who reported a spontaneous onset of labor and that their cesarean section did not occur before the onset of labor, and (2) those who reported that their labor did not begin spontaneously, that the health care provider gave them some medication to start labor, and that their cesarean section did not occur before the onset of labor. In both countries, results for this more refined definition show slight improvements to validity, and small but opposing changes to the IF. In Ghana, the IF improved from 1.18 to 1.15 and, in the Dominican Republic, the IF increased from 1.07 to 1.11. The validity of the individual question on labor induction showed very low sensitivity and high specificity in both countries. Sensitivity of reporting on spontaneous onset of labor was 84% and 89% in Ghana and the Dominican Republic, respectively. Specificity was 70% in Ghana and 51% in the Dominican Republic.

Overall validity assessed by AUC estimates show that in Ghana the indicator on emergency cesarean section by decision time had the highest validity (0.80), followed by emergency cesarean section by the operation time (0.79). The indicators tested in the Dominican Republic had moderate validity, ranging between 0.65 and 0.70, with the exception of induction of labor, which was very low (0.50).

### Exploring Accurate Reporting of Emergency Cesarean Section Status

Unadjusted odds ratios (ORs) showing the association between accurate reporting of emergency cesarean and women’s age, education, and gravidity are presented in Table 3. In Ghana, women who were referred were half as likely to report accurately on the emergency status of their cesarean section (defined by decision time) as compared to non-referrals (OR: 0.49, 95% CI 0.29–0.83, *p* = 0.009). Although there was a positive trend between emergency cesarean section (defined by decision time) and age and education, neither association was statistically significant. None of the associations with emergency cesarean section defined by operation time were statistically significant. In contrast, in the Dominican Republic there was a negative and statistically significant relationship between accurate reporting of emergency cesarean section defined by decision time and gravidity and age and between emergency cesarean section defined by operation time and age.

**Table 3:** Unadjusted odds of accurately reporting emergency cesarean section using two definitions in the Ghana and Dominican Republic samples.

	Decision Time for Cesarean Section			Operation Time for Cesarean Section(Single Question)		
	Odds Ratio	95% ConfidenceInterval	*p*-Value>|z|	Odds Ratio	95% ConfidenceInterval	*p*-Value>|z|
**Ghana (N = 659)**						
**Age**						
≤24	1.00			1.00		
25–34	1.29	0.77–2.16	0.33	0.85	0.51–1.43	0.55
≥35	1.81	0.97–3.39	0.06	1.01	0.56–1.83	0.97
**Education**						
None	1.00			1.00		
Primary	1.69	0.73–3.91	0.22	1.00	0.43–2.31	0.98
Secondary	1.65	0.88–3.09	0.12	0.94	0.49–1.83	0.87
University	2.14	0.95–4.83	0.07	0.92	0.42–2.02	0.84
**Gravidity**						
1st	1.00			1.00		
2nd	1.01	0.63–1.64	0.96	1.02	0.65–1.60	0.93
3rd	1.11	0.64–1.95	0.71	0.82	0.49–1.36	0.44
4th	1.12	0.48–2.95	0.69	1.71	0.67–4.34	0.26
**Referral**						
No	1.00			1.00		
Yes	0.49	0.29–0.83	0.01	1.39	0.92–2.10	0.115
**Dominican Republic (N = 1,531)**						
**Age**						
≤24	1.00			1.00		
25–34	0.77	0.62–0.96	0.02	0.75	0.58–0.95	0.019
≥35	0.65	0.43–1.00	0.05	0.61	0.39–0.96	0.034
**Education**						
None	1.00			1.00		
Primary	1.03	0.45–2.35	0.95	0.64	0.23–1.76	0.38
Secondary	1.36	0.60–3.07	0.46	0.79	0.29–2.14	0.64
University	1.78	0.76–4.15	0.18	0.71	0.25–1.98	0.51
**Gravidity**						
1st	1.00			1.00		
2nd	0.42	0.32–0.55	0.00	0.90	0.66–1.22	0.49
3rd	0.40	0.31–0.53	0.00	0.78	0.58–1.03	0.08
4th	0.34	0.24–0.51	0.00	0.70	0.46–1.06	0.09
**Referral**						
No	1.00			1.00		
Yes	0.91	0.72–1.15	0.41	0.94	0.73–1.23	0.67

## Discussion

Given the demand for more in-depth information on cesarean section, this study validated women’s self-report of emergency cesarean section using two definitions in two countries. Diverse populations were sought to increase generalizability and to identify survey questions, which could be recommended for use in surveys in large-scale survey programs. Although both of the study sites were referral facilities in capital cities, Ghana represents settings similar to others in much of sub-Saharan Africa and elsewhere with low skilled attendance at birth, very low population-based cesarean section rates, and high maternal mortality. In contrast, the Dominican Republic is a country with nearly universal skilled attendance at birth, and therefore high population and facility-based cesarean section rates, yet one of the highest maternal mortality ratios in Latin America.

Results from this study support the premise that self-reporting on cesarean section is valid. Although 100% of women reported that they had undergone a cesarean section, and self-report on previous cesarean section showed excellent results in both populations, validation for both of these questions would require that the question also be asked of women delivering vaginally. Nonetheless, these results, coupled with the high sensitivity and specificity for cesarean section indicator observed in the study from China in this Collection [19], increase our confidence in the widely available survey data on self-reported cesarean section.

Results from Ghana for validity and the IF for emergency cesarean section defined by decision time are promising. The poor sensitivity results for this indicator in the Dominican Republic compelled us to consider explanations with our local collaborators. On further exploration, it was discovered that this discrepancy was probably due to poor documentation of decisions during antenatal care and the practice in the delivery ward of not checking the antenatal clinical history even though most of the women who delivered at the facility also attended the antenatal clinic there. This suggests that it is likely that women’s reports are more accurate than medical records for this specific question. Validity of responses for emergency cesarean section defined by operation time in Ghana was less favorable than by decision time. In the Dominican Republic, the IF for the definition based on operation time was better than that for decision time, though with a specificity of less than 60%. The three-question approach did not improve results in either country; therefore our results do not justify the more demanding data requirements for the three-question definition relative to the one question approach. The validation results for the individual question on induced labor, of interest to maternal health planners independent of their role in identifying emergency cesarean section, cannot be recommended based on these results. However, it could possibly be improved via experimentation with different formulations of the questions.

It is important to note that the IF in our analyses was used as a measure of indicator quality and not as an adjustment factor for population-based survey results. Furthermore, there were no strong or consistent associations between women’s characteristics and accurate reporting on emergency cesarean section that could be used to adjust survey-based results.

The study has a number of limitations. First, the quality of the validation reference standard was not consistently high due to different registry systems at the hospitals, as can be observed in the Dominican Republic results. Second, this validation study does not fully replicate the conditions in the DHS and MICS surveys, because our recall period was a few days, compared to up to five years in some surveys. However, given that emergency cesarean section is a surgical intervention, we hypothesize that women are likely to remember the event and crucial circumstances surrounding it [20]. Third, even though we conducted the study in two contrasting countries, both study hospitals were tertiary care facilities in urban areas serving populations with greater access to care than in rural areas. Also, it should be noted that among the women who had cesarean sections in the Dominican Republic study, only 52% were included in the final analysis due to women who left the hospital before they could be interviewed. Given that the median duration of hospital stay across the entire Dominican Republic sample was one day, it is unlikely that this loss to follow-up biased the sample toward women with less complicated pregnancies.

Population-based cesarean section rates are essential but insufficient information for health care planners, particularly in countries without adequate routine health information systems to provide in-depth health facility-based cesarean-related data. The inadequacy of the cesarean section rate alone (without the proportion of emergency operations) is particularly acute in countries where the rate falls between 5% and 10%. In these settings, as the cesarean section rate increases, the poorest women may still not have access to life-saving delivery by cesarean section. However, the emergency cesarean section trends should be interpreted cautiously in settings such as Brazil, where cesarean sections are almost universal among certain sub-populations. [21] Although low-income countries should strive to establish robust routine health information systems which permit national-level monitoring, given current challenges, health care planners will need to rely on national surveys for the foreseeable future. Given our reliance on survey-based indicators, the most important aspect of data quality will vary by the purpose and use of the indicator. Although highly valid data are preferred for all purposes, highly sensitive and specific data are required for individual level analyses, whereas an IF near equality is sufficient for monitoring trends.

The results presented here are promising but insufficient to promote inclusion of the questions supporting the two definitions of emergency cesarean section into international survey program questionnaires. Further research on this indicator is warranted. Such studies should (1) confirm the accuracy of facility-based data on time of decision to operate in advance of data collection, (2) extend the recall period to be comparable to that of population-based surveys, and (3) based on results from the Mozambique validation study in this collection [22], allow for 50% loss to follow-up in sample size estimation to account for the extended recall period. Furthermore, qualitative research could lead to refined formulation of certain questions such as induction of labor, and potentially improve the validity of these additional indicators.

## Supporting Information

Text S1 Exit interview in Spanish.
(DOC)

## Abbreviations

AUC: area under the receiver operating characteristic curve
DHS: Demographic and Health Survey/s
IF: inflation factor
IQR: interquartile range
MICS: Multiple Indicator Cluster Survey/s
OR: odds ratio
