# Supplementary material for: Measuring Coverage in MNCH: Validating Women’s Self-Report of Emergency Cesarean Sections in Ghana and the Dominican Republic
Source: PLoS One. 2013 May 7;8(5):e60761. doi: 10.1371/journal.pone.0060761 (PMC3646217; doi:10.1371/journal.pone.0060761)
Supplement: Text S1 — Exit interview in Spanish. (DOC) [file pone.0060761.s001.doc]

**ENTREVISTA DE SALIDA PARA MUJERES**

| **#** | **PREGUNTAS** |  | **OMITAR** |
| --- | --- | --- | --- |
|  | **ANTECEDENTES** |  |  |
|  | ¿Cuál es su edad? |  |  |
| **1a.** | ¿Cuál es su fecha de nacimiento? (no contesta si la paciente no sabe) | | día | mes | año | | --- | --- | --- | |  |  |  | |  |
|  | ¿Cuál es su religión? | Católico..………………………………………..……………0  Evangélico…………………………………………..………1  Testigo de Jehová..……………………………...…….…2  Adventista…………………………………………………..3  Otra, especifique_________________________________4  Ninguna………………………………………………………5 |  |
|  | ¿Con qué nacionalidad se identifica? | Dominicana…………………………………………………0  Haitiana………………………………………………………1  Otra………………………………………………………….…2  Otra, especifique_________________________________8 |  |
|  | ¿Cuál es su estado civil? | Casada/Unión civil.…...…………………………….…..0 Divorciada/Separada…………………………………..1  Soltera..…………………………………………………..…..2 |  |
|  | ¿Qué nivel de estudios ha obtenido? | Ninguno.………………………..…...……………………….0  Primarios…………………………..………………………..1 Secundarios……………….………………………………..2 Universitarios…………….……………………………….3 Tercer…………………………………….…………………..4 |  |
|  | ¿En qué parte del país (provincia) vive? | Distrito Nactional  Santo Domingo  Azua  Baoruco  Barahona  Dajabón  Duarte  El Seibo  Elías Piña  Espaillat  Hato Mayor  Hermanas Mirabal  Independencia  La Altagracia  La Romana  La Vega  María Trinidad Sánchez  Monseñor Nouel  Montecristi  Monte Plata  Pedernales  Peravia  Puerto Plata  Samaná  San Cristóbal  San José de Ocoa  San Juan  San Pedro de Macrorís  Sánchez Ramírez  Santiago  Santiago Rodríguez  Valverde  Otra |  |
|  | Especifique el municipio de Santo Domingo: | Santo Domingo Este  Boca Chica  Los Alcarrizos  Pedro Brand  San Antonia de Guerra  Santo Domingo Norte  Santo Domingo Oeste |  |
|  | Para otro, especifique: (para extranjeras especifique el país) |  |  |
|  | ¿Cómo describe el lugar donde vive? | Zona urbana………………………………………………..0  Zona rural………………………………………………...…1  Desconocido…………………………………….………....9 |  |
|  | **SALUD REPRODUCTIVA** |  |  |
|  | Ahora me gustaría preguntarle acerca de todos los embarazos que ha tenido en su vida. ¿Alguna vez ha dado a luz? | SÍ………………………………………………………………..0  NO………………………………………………………..…….1 |  |
|  | ¿Cuántos hijos varones que ha dado a luz están vivos?  ¿Cuántos hijas (mujeres) que ha dado a luz están vivas? | 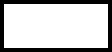  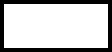 |  |
|  | ¿Ha dado a luz a un hijo o una hija que nació vivo y luego murió? (SI RESPONDE NO: pregunte si tuvo un bebé que lloró al nacer y luego falleció) | SÍ………………………………………………………………..0  NO………………………………………………………..…….1 |  |
|  | ¿Cuántos hijos varones fallecieron de esa manera?  ¿Cuántas hijas (mujeres) fallecieron de esa manera? | 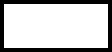  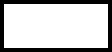 |  |
|  | ¿Ha perdido algún embarazo? Puede ser espontáneo o inducido (inducido es cuando usted u otra persona tuvieron que hacer algo para terminar el embarazo). | SÍ………………………………………………………………..0  NO………………………………………………………..…….1 |  |
|  | ¿Cuántos embarazos ha perdido? | 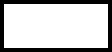 |  |
|  | Hay mujeres que tienen nacidos muertos, que dan a luz tarde en el embarazo a un bebé muerto. ¿Ha tenido algún nacido muerto? | SÍ………………………………………………………………..0  NO………………………………………………………..…….1 |  |
|  | ¿Cuántos nacidos muertos ha tenido en su vida? | 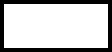 |  |
|  | Antes de este embarazo, ¿había tenido alguna cesárea? | SÍ………………………………………………………………..0  NO………………………………………………………..…….1 |  |
|  | Si contesta SÍ, para antes de este embarazo, ¿cuántas cesáreas había tenido? | 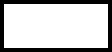 |  |
|  | ¿Ha tenido otras cirugías en su área pélvica? | SÍ………………………………………………………………..0  NO………………………………………………………..…….1 |  |
|  | Si contesta SÍ, ¿qué cirugía fue? |  |  |
|  | **PARTO ACTUAL** |  |  |
|  | Ahora le voy a preguntar sobre su parto reciente. ¿Había programado dar a luz en la Maternidad de la Altagracia? | SÍ………………………………………………………………..0  NO………………………………………………………..…….1 |  |
|  | Si contesta NO, ¿dónde había programado dar a luz? |  |  |
|  | ¿Fue trasladada de otro centro? | SÍ………………………………………………………………..0  NO………………………………………………………..…….1 |  |
|  | Si contesta SÍ, ¿de dónde? | ____________________________________________________ |  |
|  | ¿Cuál fue la razón del traslado? |  |  |
|  | ¿Que tipo de parto ha tenido en la Maternidad de la Altagracia? | Vaginal……………………………………………………..…0  Fórceps/Aspiradora..………………………………..…1  Parto por cesárea………………………………………...2 |  |
|  | ¿Cuál fue la razón de su cirugía durante su parto? Elija la razón(es) que aplica(n) mejor a su situación. | El médico/la enfermera me dijo que tenía que hacerlo. No sé la razón.………………………..………0  Estaba sangrando………………………………...……...1  El bebe quedó atrapado……………………………………………..………2  Estuve en trabajo de parto por mucho tiempo3  El bebé no estaba en buena posición………...…..4  Tenía una enfermedad ………………………...……...5  Tuve ruptura de matriz/útero …………..……...6  Tuve problemas con el bebé………………………...7  Fue mi primer embarazo y me dijeron que fue por mi edad……………………………………………8  Lo solicité……………………………………….…………..9  Desconocido….……………………………….………….10  Otro (especifique) _____________________________11 |  |
|  | SI PIDIÓ LA CESÁREA (si no, salte la pregunta) ¿por qué pidió usted la cesárea? |  |  |
|  | ¿Cuándo se tomó la decisión de su cesárea/cirugía? | Durante el embarazo/ los chequeos prenatales…………………0  Antes de empezar el trabajo de parto………..…1  Después que empezó el trabajo de parto …………2  Desconocido…………………………………………..…..9 |  |
|  | ¿De quién fue la idea de que usted tuviera una cesárea? Por favor, seleccione la opción que lo describe mejor. | El médico dijo que tenía que hacerlo………….…0  Yo solicité la cesárea………….....................................1  Otro (especifique)  __________________________________________________­­__8  Desconocido…………………..……………………….…..9 |  |
|  | ¿Quien le informó que iba a tener una operación/cesárea? (la primer vez) | Médico………………………………….…………………….0  Residente………………………...………………………….1  Estudiante de medicina ……………….……………...4  Enfermera.………………………………..………………...2  Partera…….…………………………………..……………..3  Médico interno……………………………………………4  Familiar……………………………………………………....5  Nadie…………………………………………………………..6  Otro………………………………………………………….…7  Para Otro, especifique por favor_________________ |  |
|  | ¿Tuvo un embarazo único? | SÍ………………………………………………………………..0  NO………………………………………………………..…….1 |  |
|  | ¿Tuvo un parto gemelar? | SÍ………………………………………………………………..0  NO………………………………………………………..…….1 |  |
|  | Antes del nacimiento, ¿cuál fue la posición del bebé?  Para los partos gemelares, ¿cuál fue la posición del segundo bebé? | Cabeza abajo……………………………………………….0  Pies abajo……………...…………………………………....1  Cualquier otra posición ………………………………2  Desconocido………………….…………………………….9 |  |
|  | ¿Empezó la labor de parto por si misma/ espontáneamente? | SÍ………………………………………………………………..0  NO………………………………………………………..…….1  Desconocido…………………………………….………....9 |  |
|  | ¿Algún proveedor de salud le dio algún medicamento o goteo para INICIAR su labor de parto? | SÍ………………………………………………………………..0  NO………………………………………………………..…….1  Desconocido……………………….……………………....9 |  |
|  | ¿Un proveedor de salud le dio un medicamento o un goteo para ACELERAR su labor de parto? (reforzar sus contracciones)? | SÍ………………………………………………………………..0  NO………………………………………………………..…….1Desconocido…………………………………………….....9 |  |
|  | ¿Le hicieron una cesárea ANTES de que empezó su trabajo de parto? | SÍ………………………………………………………………..0  NO………………………………………………………..…….1  Desconocido……………………..………………………...9 |  |
|  | ¿Cuántas semanas de embarazo tenía cuando dió a luz? | __________semanas  Desconocido…………………….…………………..……..9 |  |
|  | ¿Cuántos meses de embarazo tenía? | ___________meses  Desconocido……………………………………..………..9 |  |
|  | ¿Nació el bebé prematuro? ¿Fue antes de la fecha prevista del parto? | SÍ………………………………………………………………..0  NO………………………………………………………..…….1  Desconocido……………………………………………....9 |  |
|  | ¿Nació a tiempo el bebé (a término)? | SÍ………………………………………………………………..0  NO………………………………………………………..…….1  Desconocido……………………………………………....9 |  |
|  | ¿Preferiría tener una cesárea en embarazos futuros? | SÍ………………………………………………………………..0  NO………………………………………………………..…….1  Desconocido……………………………………………....9 |  |
|  | ¿Por qué? |  |  |
| **END** | Muchas gracias por su tiempo y por hablar con nosotros. | | |

Nombre de entrevistador:

Fecha de entrevista:

| día | mes | año |
| --- | --- | --- |
|  |  |  |
